# Acetylshikonin Induces Apoptosis in Human Colorectal Cancer HCT-15 and LoVo Cells via Nuclear Translocation of FOXO3 and ROS Level Elevation

**DOI:** 10.1155/2021/6647107

**Published:** 2021-04-13

**Authors:** Heui Min Lim, Jongsung Lee, Myeong Jin Nam, See-Hyoung Park

**Affiliations:** ^1^Department of Biological Science, Gachon University, Seongnam 13120, Republic of Korea; ^2^Department of Integrative Biotechnology, Sungkyunkwan University, Suwon 16419, Republic of Korea; ^3^Department of Bio and Chemical Engineering, Hongik University, Sejong 30016, Republic of Korea

## Abstract

Acetylshikonin, a naphthoquinone, is a pigment compound derived from Arnebia sp., which is known for its anti-inflammatory potential. However, its anticarcinogenic effect has not been well investigated. Thus, in this study, we focused on investigating its apoptotic effects against HCT-15 and LoVo cells, which are human colorectal cancer cells. MTT assay, cell counting assay, and colony formation assay have shown acetylshikonin treatment induced cytotoxic and antiproliferative effects against colorectal cancer cells in a dose- and time-dependent manner. DNA fragmentation was observed via terminal deoxynucleotidyl transferase dUTP nick end labeling (TUNEL) assay. Also, the increase of subG1 phase in cell cycle arrest assay and early/late apoptotic rates in annexin V/propidium iodide (PI) double staining assay was observed, which indicates an apoptotic potential of acetylshikonin against colorectal cancer cells. 2′,7′-Dichlorofluorescin diacetate (DCF-DA) staining was used to evaluate reactive oxygen species (ROS) generation in acetylshikonin-treated colorectal cancer cells. Fluorescence-activated cell sorting (FACS) analysis showed that acetylshikonin induced an increase in reactive oxygen species (ROS) levels and apoptotic rate in a dose- and time-dependent manner in HCT-15 and LoVo cells. In contrast, cotreatment with N-acetyl cysteine (NAC) has reduced ROS generation and antiproliferative effects in colorectal cancer cells. Western blotting analysis showed that acetylshikonin treatment induced increase of cleaved PARP, *γ*H2AX, FOXO3, Bax, Bim, Bad, p21, p27, and active forms of caspase-3, caspase-7, caspase-9, caspase-6, and caspase-8 protein levels, while those of inactive forms were decreased. Also, the expressions of pAkt, Bcl-2, Bcl-xL, peroxiredoxin, and thioredoxin 1 were decreased. Furthermore, western blotting analysis of cytoplasmic and nuclear fractionated proteins showed that acetylshikonin treatment induced the nuclear translocation of FOXO3, which might result from DNA damage by the increased intracellular ROS level. This study represents apoptotic potential of acetylshikonin against colorectal cancer cells via translocation of FOXO3 to the nucleus and upregulation of ROS generation.

## 1. Introduction

Colorectal cancer is a malignancy that occurs in the colon or the rectum. Both forms may simply form as independent colon or rectal cancers, but they often merge due to their common features [1]. It was reported that colorectal cancer is the third leading cause of cancer death and fourth most common cancer diagnosed in the world [2]. Also, colorectal cancer is the second most leading cause of cancer death in the United States, when men and women are combined. The incidence of colorectal cancer has been increasing worldwide, remarkably in developing countries [3]. It was suggested that age, genetic, and environmental factors are the main factors on development of colorectal cancer. Well-known risk factors of colorectal cancer include obesity, red meat and processed meat, smoking habit, alcohol consumption, history of abdominal radiation, and familiar histories [4]. There are also protective factors that have correlation with decreasing colorectal cancer incidence. Regular physical activity and intake of more fruits, vegetables, and high-fiber diet had evidently reduced development of colorectal cancer after diagnosis [5]. Nowadays, colorectal cancer is transformed to increasingly curable disease due to advanced diagnostics such as routine colonoscopies [6, 7]. However, surgical resection remains as the only option to cure colon and rectal cancers [8]. Therefore, identifying the candidate matter as a curative option for colorectal cancer is of high value.

Acetylshikonin is one of the naturally produced shikonin derivatives, which are pigment components originated from Lithospermum erythrorhizon roots [9]. Lithospermum erythrorhizon is a Chinese medicinal herb that has various functions such as inhibition of transcription activation in human tumor cells and treatment of wounded skins via promoting inflammatory effects, granulation tissue formation, and inhibition of angiogenesis in vitro and in vivo [10, 11]. The effects of acetylshikonin against inflammatory diseases and tumor cells via inhibition of vascular endothelial growth factor- (VEGF-) induced angiogenesis were well investigated formerly in previous studies [12, 13]. There were also a number of studies which investigated anticarcinogenic effects of acetylshikonin in human cancer cells [14, 15]. However, the apoptotic mechanism, of acetylshikonin against colorectal cancer cells is still not thoroughly investigated.

Forkhead box O-3 (FOXO3) is a member of the human transcript factor forkhead box (FOX) gene family [16], which exhibits associations with longevity in human populations and its genetic polymorphisms [17]. FOXO3 is a regulator protein responsible for oxidative stress resistance, metabolism, cell cycle, and cellular apoptosis [18]. FOXO3 protein levels can be regulated by degradation, transcription, and mutation in FOXO genes [19]. Various protein modification mechanisms are involved in regulation of FOXO3. Downregulation of FOXO3 can be mediated by phosphorylation of FOXO3 protein by protein kinase B (Akt) [20, 21] or polyubiquitination led by SIRT1/SIRT2-induced deacetylation of FOXO3 [22]. Conversely, increased transcriptional activity of FOXO3 can be induced by phosphorylation of active protein kinases such as 5′-AMP-activated protein kinase (AMPK), Akt, and glycogen synthase kinase-3 (GSK-3) [23, 24]. Increased FOXO3 activity induces apoptotic signaling via facilitating the expressions of proapoptotic Bcl2 family members or stimulation of tumor necrosis factor (TNF) family [25, 26]. Recent studies have revealed the association between FOXO3 and cellular apoptosis of tumor cells [26–28], which suggest that the further investigation of FOXO3-related apoptotic mechanisms in tumor cells will bring about a promising strategy on developing therapeutic alternatives.

In this study, we hypothesized that acetylshikonin induces apoptosis in colorectal cancer via activation of FOXO3 expression, thus the downstream apoptotic pathways. To verify the hypothesis, we performed various apoptosis analyses to detect induction of apoptosis and western blotting to investigate alterations of pro- and antiapoptotic proteins, which are located up- or downstream of FOXO3.

## 2. Materials and Methods

### 2.1. Chemical Reagents and Antibodies

Acetylshikonin was purchased from ChemFaces (CheCheng Rd. WETDZ, Wuhan, China) and dissolved in dimethyl sulfoxide (DMSO, Sigma, St. Louis, MO, USA). 20 mM stock solutions of preparation were stored at -20°C. N-acetyl cysteine (NAC) was purchased from Sigma (St. Louis, MO, USA). ERK inhibitor (U0126) and JNK inhibitor (SP600125) were purchased from Cell Signaling Technology (Danvers, MA, USA) and p38 inhibitor (PD169316) was purchased from Sigma (St. Louis, MO, USA). MAPK inhibitors are treated in 10 *μ*M for experiment. Caspase-3 (1 : 1000 dilution), caspase-7 (1 : 1000 dilution), caspase-6 (1 : 1000 dilution), caspase-9 (1 : 1000 dilution), cleaved caspase-3 (1 : 1000 dilution), cleaved caspase-7 (1 : 1000 dilution), cleaved caspase-6 (1 : 1000 dilution), cleaved caspase-8 (1 : 1000 dilution), cleaved caspase-9 (1 : 1000 dilution), Akt (1 : 1000 dilution), pAkt (1 : 1000 dilution), ERK (1 : 1000 dilution), pERK (1 : 1000 dilution), PARP (1 : 1000 dilution), cleaved-PARP (1 : 1000 dilution), Bim (1 : 1000 dilution), Bcl-2 (1 : 1000 dilution), Bad (1 : 1000 dilution), pBad (1 : 1000 dilution), p21 (1 : 1000 dilution), p27 (1 : 1000 dilution), H2aX (1 : 1000 dilution), and *λ*H2aX (1 : 1000 dilution) primary antibodies were purchased from Cell Signaling Technology (Danvers, MA, USA). *β*-Actin (1 : 2000 dilution), caspase-8 (1 : 500 dilution), JNK2 (1 : 1000 dilution), pJNK (1 : 1000 dilution), p38 (1 : 500 dilution), and pp38 (1 : 500 dilution) primary antibodies were obtained from Santa Cruz Biotechnology (Santa Cruz, CA, USA). Bcl-xL (1 : 1000 dilution) and FOXO3 (1 : 1000 dilution) were purchased from Youngin Frontier (Seoul, Korea). Goat anti-mouse (1 : 3000 dilution) and goat anti-rabbit (1 : 3000 dilution) horseradish secondary antibodies were purchased from Cell Signaling Technology (Danvers, MA, USA) [29].

### 2.2. Cell Culture

Human colorectal carcinoma HCT-15 and LoVo cells (from ATCC, Manassas, VA, USA) were maintained in RPMI media, and human colon fibroblast CCd-18Co (from KCLB, Seoul, Korea) was maintained in DMEM; both are supplemented with 10% fetal bovine serum (FBS) and 1% antibiotics (streptomycin/penicillin) at standard conditions (37°C in a humidified incubator containing 5% CO_2_ in air). The cells were harvested at 70~80% of confluency and reseeded for expansion and experiments [30].

### 2.3. MTT Cell Viability Assay

A 200 *μ*L aliquot of HCT-15 and LoVo cells (5 × 10^3^ cells in media) was added to each well of 96-well plates and incubated for 24 h under standard conditions. After 24 h of incubation, the cells were treated with acetylshikonin at a concentration gradient of 0 to 10 *μ*M and incubated for 24, 48, and 72 h for the time-dependent response assay. After 24, 48, and 72 h of treatment, a 20 *μ*L MTT dye solution (5 mg/mL in phosphate buffer) was added to each well and the incubation for 2 h. After all the media were removed, formazan was solubilized in 200 *μ*L DMSO, and light absorbance was measured at a wavelength of 570 nm using a microplate reader [31].

### 2.4. Cell Counting Assay

HCT-15 and LoVo cells (2 × 10^4^/well) were seeded in 6-well plates and incubated for 18 h. After 18 h of incubation, cells were treated with concentrations 0, 1.25, 2.5, and 5 *μ*M of acetylshikonin and DMSO as control vehicle for 0, 24, and 72 h. Each day, cell numbers were counted by using a hemocytometer [32].

### 2.5. Colony Forming Assay

HCT-15 and LoVo cells (0.5 × 10^3^/well) were seeded in 6-well plates and for 24 h. After 24 h of incubation, cells were treated with 1.25 *μ*M of acetylshikonin and DMSO as control vehicle for 24 h. After 24 h of treatment, the cells were cultured in fresh media for 12 days. PBS washing was performed twice for 3 min, and cells were fixed with 4% formaldehyde solution for 20 min at 4°C. Followed by fixation, the cells were washed twice with PBS for 3 min and stained with 1% crystal violet (Sigma, St. Louis, MO, USA) solution for 30 min at room temperature (25°C) [33].

### 2.6. Annexin V/PI Staining Assay

Annexin V-FITC apoptosis staining/detection kit (ab14085, Abcam, Cambridge, UK) was used to detect cellular apoptosis. HCT-15 and LoVo cells (4 × 10^5^ cells) were seeded in 6-well plates and cultured for 24 h. After 24 and 48 h of treatment with acetylshikonin (0, 1.25, 2.5, and 5 *μ*M), each well was washed twice with PBS and harvested using trypsin-EDTA (Sigma, St. Louis, MO, USA). The supernatant was removed after centrifugation, suspended in 1× binding buffer (5 *μ*L of annexin V-FITC and 5 *μ*L propidium iodide added), and incubated for 5 min. Apoptotic cells were quantified by flow cytometry (Beckman Coulter, Brea, CA, USA) [33].

### 2.7. Cell Cycle Arrest

A cell cycle arrest induced by acetylshikonin in HCT-15 and LoVo cells was analyzed. The cells were collected after 24 and 48 h of treatment with acetylshikonin. Then, the cells were suspended in cold 70% ethanol and fixed at -20°C for 18 h. The fixed cells were centrifuged, and supernatants were carefully removed with a pipette. Pelleted cells were incubated with 1 mL of DNA staining solution (50 *μ*g/mL of propidium iodide and 200 *μ*g/mL of DNase-free RNase in PBS with Triton X-100 diluted to 0.2% for permeability) for 30 min. An FC 500 series cytometer (Beckman Coulter) was used for acquisition and analysis. Flow cytometric data were organized using the CXP program (Beckman Coulter) [33].

### 2.8. TUNEL Assay

DNA fragmentation was detected via terminal deoxynucleotidyl transferase- (TdT-) mediated dUTP nick end labeling (TUNEL) assay using the fluorometric TUNEL system (G3250, Promega, Madison, WI, USA). HCT-15 and LoVo cells were seeded in 6-well plates at a density of 4 × 10^5^ per well and cultured for 24 h. After treatment with acetylshikonin (0, 1.25, 2.5, and 5 *μ*M), cells were fixed with 4% formaldehyde solution for 25 min at 4°C and permeabilized using Triton X-100, diluted to 0.5% in PBS for 10 min. Apoptotic cells were treated with 25 *μ*L TdT enzyme buffer. All cells were then stained using Hoechst stain solution (Sigma, St. Louis, MO, USA). Fluorescence-labeled damaged DNA strands were visualized using a fluorescence microscope (Nikon Eclipse TE 2000-U, Tokyo, Japan). Images were taken at 200x magnification [34].

### 2.9. DCF-DA Staining

Intracellular ROS generation in acetylshikonin-treated colorectal cancer cells was analyzed using a 20,70-dichlorofluorescein diacetate (DCF-DA) cellular ROS detection assay kit. HCT-15 and LoVo cells were treated with 0, 1.25, 2.5, and 5 *μ*M acetylshikonin for 6 h. Cells were also cotreated with or without 5 mM N-acetyl cysteine (NAC) in control and 5 *μ*M of acetylshikonin. Treated cells and supernatants were collected using trypsin-EDTA. The collected cells were pelleted and incubated at room temperature with 25 *μ*M DCF-DA solution for 30 min. Intracellular ROS generation in treated cells was analyzed using flow cytometry. An FC 500 series cytometer (Beckman Coulter) was used for flow cytometric analysis. Flow cytometric data were organized using the CXP program (Beckman Coulter) [35].

### 2.10. Western Blot Analysis

After 24 h of treatment with acetylshikonin (0, 1.25, 2.5, and 5 *μ*M), total proteins from HCT-15 and LoVo cells were extracted using RIPA buffer (Sigma, St. Louis, MO, USA) with protease and phosphatase inhibitors, PMSF (phenylmethylsulfonyl fluoride) (Sigma, St. Louis, MO, USA). Proteins were loaded on 12% SDS-PAGE gels and blotted onto the polyvinylidene difluoride (PVDF) membrane (Millipore, Billerica, MA, USA). The membranes were blocked with 3% bovine serum albumin (BSA; Bovogen, Victoria, Australia) for 30 min at room temperature (25°C) and probed with primary antibodies at 4°C overnight. The membranes were then incubated with HRP-tagged secondary antibodies for 1 h at room temperature (25°C). Protein bands were visualized by using the enhanced chemiluminescence (ECL; Gendepot, Barker, USA) and detected with ChemiDoc detection system (Bio-Rad, Hercules, CA, USA) [36].

### 2.11. Cytoplasmic and Nuclear Protein Fractionation

Cytoplasmic and nuclear proteins were separately extracted using Nuclear/Cytosol Fractionation Kit (K266, BioVision, Inc., Milpitas, CA, USA). After 12 h of treatment with acetylshikonin (0, 1.25, 2.5, and 5 *μ*M), cells were collected and washed 2 times with PBS. Supernatants were removed, and cells were resuspended in cytosol extraction buffer-A (CEB-A), vortexed for 15 s at highest setting and incubated in ice for 10 min. Ice-cold cytosol extraction buffer-B (CEB-B) was then added, vortexed 5 s at highest setting and incubated in ice for 1 min. The samples were vortexed for 5 s and centrifuged (14,000 rpm at 4°C for 5 min) to acquire cytoplasmic fraction. The remaining pellets were washed 2 times with PBS, and nuclear extraction buffer (NEB) was added, vortexed 15 s at highest setting and incubated in ice for 10 min. After the vortex and incubation procedure was repeated four times, the samples were centrifuged (14,000 rpm at 4°C for 10 min) to acquire nuclear fraction. The procedure was performed according to the manufacturer's protocol.

### 2.12. Statistical Analysis

The results are expressed as the arithmetic mean + standard deviation. To compare the data between the groups, two-sided unpaired Student's *t*-test was used. Experiments were repeated three times, and the representative data were shown. A one-way ANOVA followed by the Bonferroni post hoc test was used for statistical analysis, and a *p* value of <0.05 was considered statistically significant.

## 3. Results

### 3.1. Acetylshikonin Inhibited Cell Viability and Proliferation in HCT-15 and LoVo Cells

**Figure 1:**
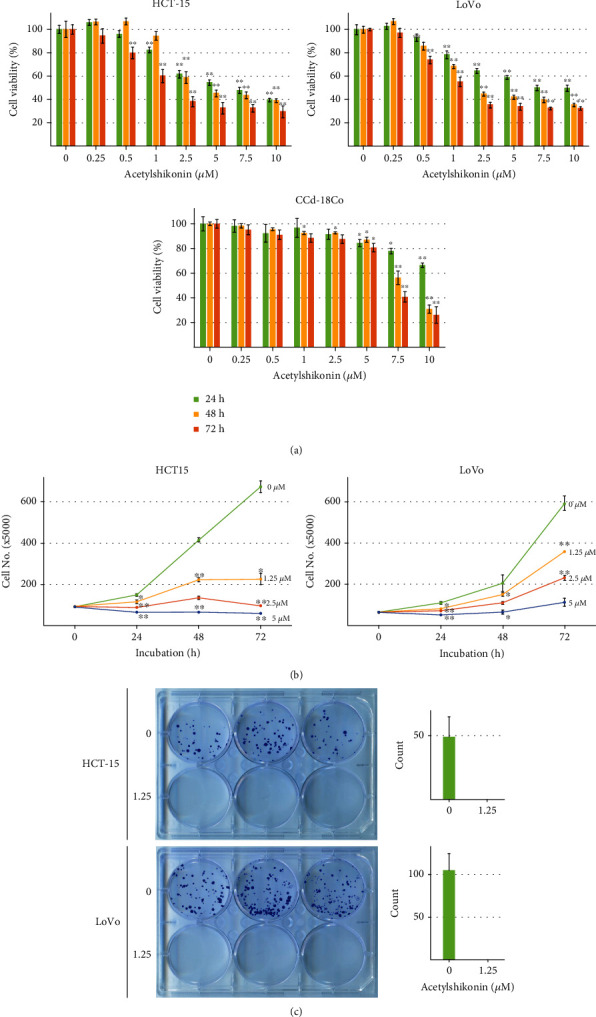
Cell viability and antiproliferative effect of acetylshikonin-treated HCT-15 and LoVo cells. (a) The cytotoxic effect of acetylshikonin on HCT-15 and LoVo cells. Human colorectal cancer cells HCT-15 and LoVo cells, and normal human colon fibroblast CCd-18Co cell were treated with acetylshikonin with concentrations ranging from 0 to 10 *μ*M for 24, 48, and 72 h. The cell viability was analyzed by MTT assay. (b) Acetylshikonin inhibits cell survival and proliferation of HCT-15 and LoVo cells. The cell numbers were analyzed by the cell counting assay after HCT-15 and LoVo cells (2 × 10^4^ cells/plate) were treated with acetylshikonin (0, 1.25, 2.5, and 5 *μ*M) for 0, 24, 72, and 120 h. (c) Acetylshikonin suppresses the colony-forming ability of HCT-15 and LoVo cells. The colony numbers of cells were analyzed by the colony forming assay. HCT-15 and LoVo cells (500 cells/well) were treated with acetylshikonin (1.25 *μ*M) or the control for 24 h and cultured for 12 days. The representative images of the assays are shown. The bar graphs represent a quantitation of the number of acetylshikonin-treated HCT-15 and LoVo colonies. Data are represented with the mean ± SD of triplicated results. Single and double asterisks indicate significant differences from the control cells (^∗^*p* < 0.05 and ^∗∗^*p* < 0.01, respectively).

Cell viability was analyzed by MTT assay. Dose- and time-dependent inhibition of cell viability was observed in acetylshikonin-treated cells. The result shows that acetylshikonin has a significant (*p* < 0.05) inhibitory effect from 1 *μ*M. The 50% inhibitory concentration of acetylshikonin was 5.14 *μ*M (IC50 = 5.14 *μ*M) at 24 h in HCT-15 cells and 6.41 *μ*M (IC50 = 6.41 *μ*M) at 24 h in LoVo cells. However, it showed minor cytotoxic effect against normal human colon fibroblast CCd-18Co up to 5 *μ*M (IC50 = 8.53 *μ*M at 48 h and IC50 = 7.38 *μ*M at 72 h) (Figure 1(a)). Cell counting assay and colony forming assay were performed to analyze and visually demonstrate the effect of acetylshikonin in proliferation of colorectal cancer cells. The result from cell counting assay showed decreases of cell viability in acetylshikonin-treated cells (Figure 1(b)). Furthermore, the results from colony forming assay show that the treatment of acetylshikonin had completely inhibited the proliferation of HCT-15 and LoVo cells even in the lowest concentration at low confluency of colorectal cancer cells (Figure 1(c)). These results indicate that acetylshikonin has an inhibitory effect against the cell viability of human colorectal cancer HCT-15 and LoVo cells in a dose- and time-dependent manner.

### 3.2. Acetylshikonin Induced Apoptosis in HCT-15 and LoVo Cells

**Figure 2:**
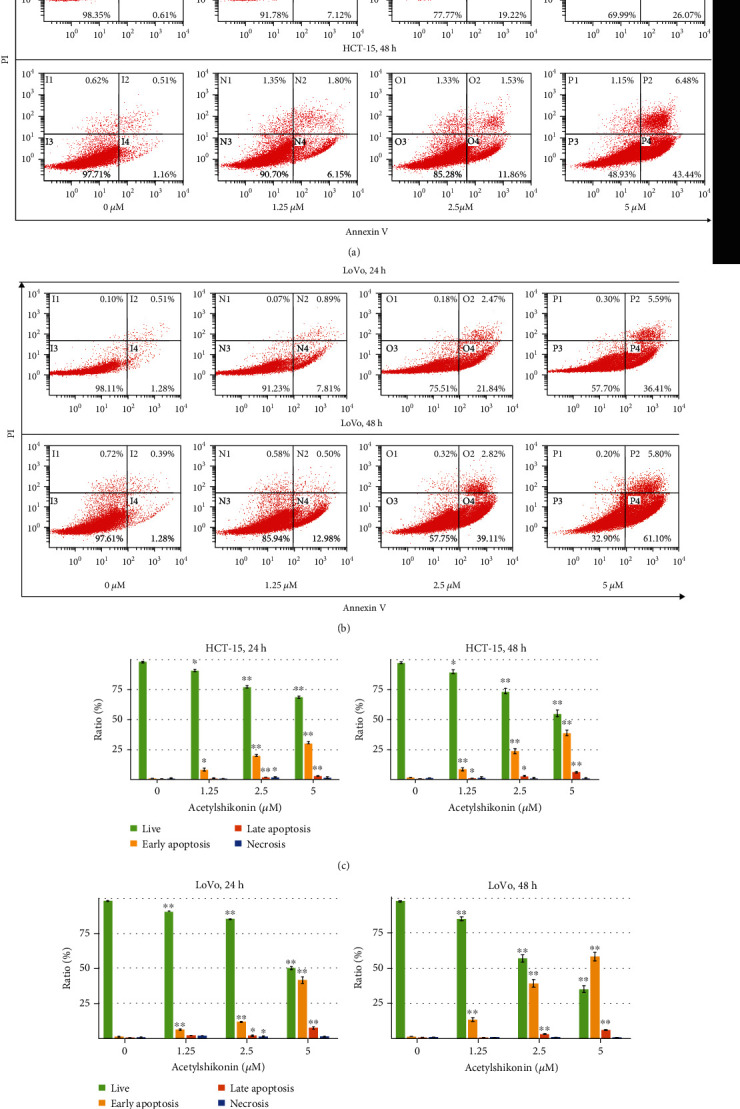
FACS analysis of annexin V/PI double-stained HCT-15 and LoVo cells. HCT-15 and LoVo cells were treated with 0, 1.25, 2.5, and 5 *μ*M of acetylshikonin for 24 and 48 h. (a, b) The treated cells were then stained with annexin V and PI for apoptotic analysis. The percentages of apoptotic cells are indicated on the plots. (c, d) Percentages of each portion in apoptotic cells were represented in bar graphs. The data represent the mean ± SD of three independent experiments. Single and double asterisks indicate significant differences from the control cells (^∗^*p* < 0.05 and ^∗∗^*p* < 0.01, respectively).

**Figure 3:**
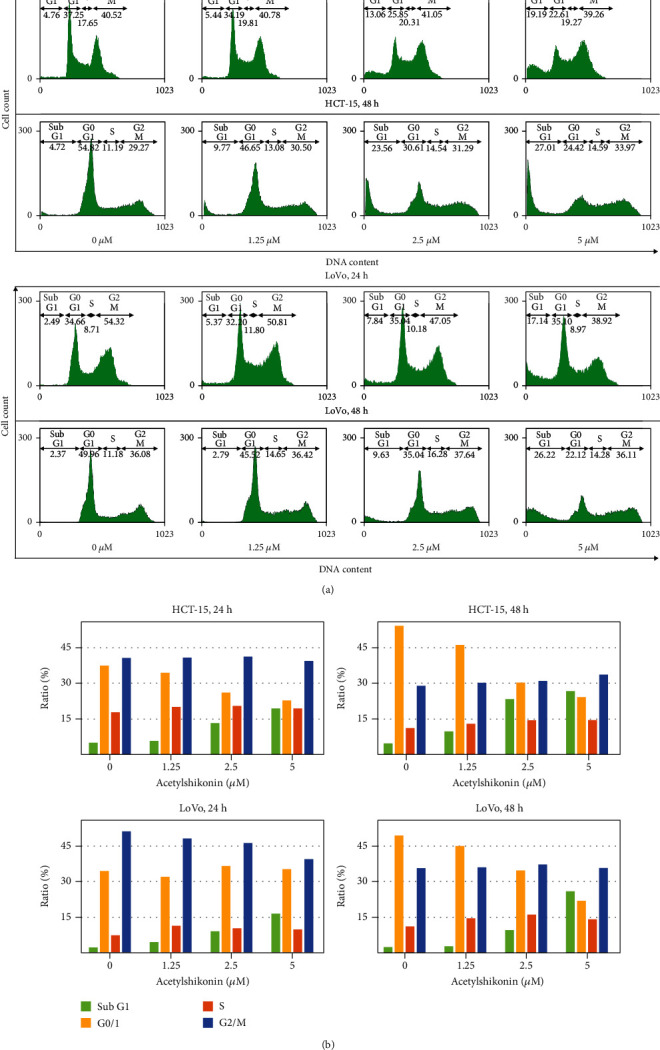
Cell cycle progression analysis of acetylshikonin-treated HCT-15 and LoVo cells. (a) Cells were treated with 0, 1.25, 2.5, and 5 *μ*M of acetylshikonin for 24 and 48 h and stained with propidium iodide (PI) for a flow cytometric analysis of DNA content. (b) Data are represented in bar graphs.

**Figure 4:**
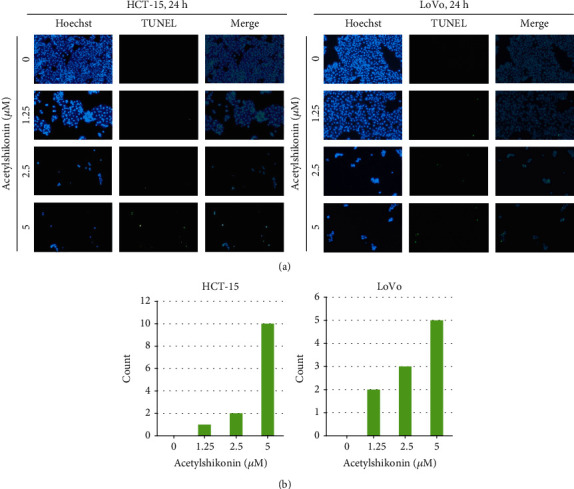
Detection of DNA fragmentations in HCT-15 and LoVo cells after 24 h treatment with 0, 1.25, 2.5, and 5 *μ*M of acetylshikonin with TUNEL assay. (a) Blue fluorescence, stained with Hoechst, shows the whole nuclei of cells, while green fluorescence, labeled with TdT, only shows fragmented DNA. Images were taken at 200x magnification. Hoechst-stained cell images and the TdT-labeled fragmented DNA images were merged to visualize the comparisons of acetylshikonin-induced DNA fragmentations in HCT-15 and LoVo cells in a dose- and time-dependent manner. (b) Graphical analysis of dyed cells.

To investigate acetylshikonin-induced apoptosis, annexin V/PI double staining assay and cell cycle arrest assay were performed using flow cytometry. The apoptotic rates of HCT-15 cells at 24 h were increased to 7.71%, 20.69%, and 28.69% (*p* = 0.0001), and at 48 h, they were increased to 7.95%, 13.39%, and 49.92% (*p* = 0.0006), respectively (Figures 2(a) and 2(c)). In LoVo cells, the apoptotic rates were increased to 8.7%, 24.31%, and 42% (*p* = 0.0003) at 24 h and they rose to 13.48%, 41.93%, and 66.9% (*p* = 0.0006) at 48 h (Figures 2(b) and 2(d)). In the cell cycle arrest assay, the result showed that the portions of subG1 phase were increased (4.76%, 5.44%, 13.06%, and 19.19% in HCT-15 and 2.49%, 5.37%, 7.84%, and 17.14% in LoVo after 24 h of treatment; 4.72%, 9.77%, 23.56%, and 27.01% in HCT15 and 2.37%, 2.79%, 9.63%, and 26.22% in LoVo after 48 h of treatment) as the concentration of acetylshikonin was increased (Figures 3(a) and 3(b)). TUNEL assay visualized the DNA damage via enzymatic labeling of free 3′-end of DNA, which is one of the features of apoptosis; it is observed that the number of TUNEL-positive cells was significantly increased in acetylshikonin-treated cells (Figures 4(a) and 4(b)). These results show that the apoptotic rates of colorectal cancer cells treated with acetylshikonin were increased in a dose- and time-dependent manner and significantly in 5 *μ*M of concentration.

### 3.3. Acetylshikonin Induced Intracellular ROS Level Elevation in HCT-15 and LoVo Cells

**Figure 5:**
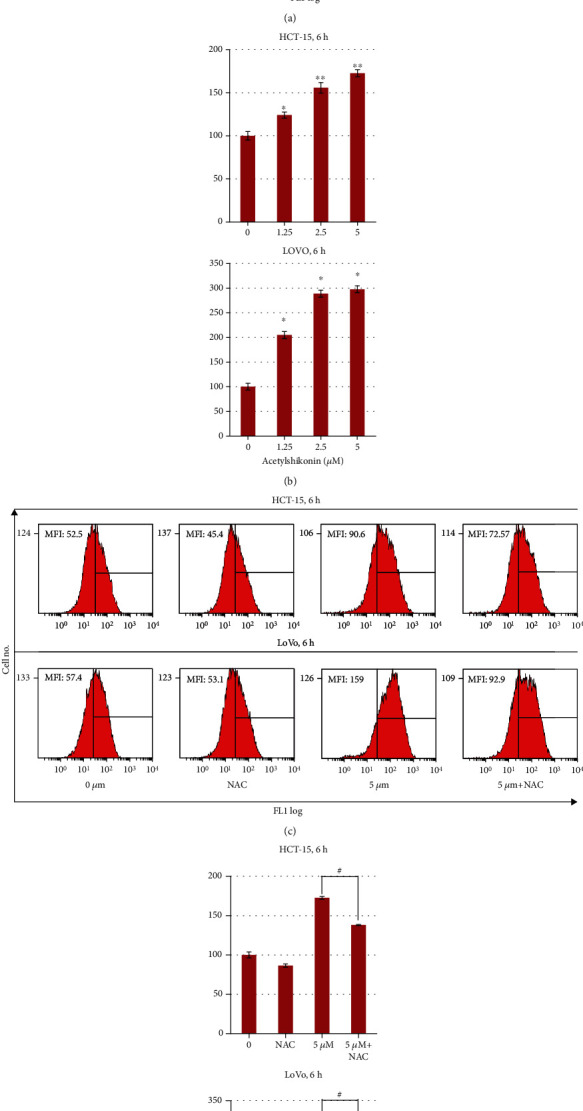
ROS generation in colorectal cancer HCT-15 and LoVo cells treated with acetylshikonin. (a, c) Intracellular ROS generation in acetylshikonin-treated HCT-15 and LoVo cells and those cotreated with NAC was measured by using DCFH-DA (10 *μ*M) and flow cytometry after 6 h of treatment. Mean fluorescence intensity (MFI) at each concentration is indicated on each plot. Mean fluorescence intensity (MFI) indicated on each plot. (b, d) Bar graph shows quantitation of the MFI. The vector control MFI was set at 100%. The data represent the mean ± SD of three independent experiments. Single and double asterisks indicate significant differences from the control cells (^∗^*p* < 0.05 and ^∗∗^*p* < 0.01, respectively). The data represent the mean ± SD of three independent experiments. Number sign indicates a significant difference of NAC-treated cells from acetylshikonin-treated cells (^#^*p* < 0.05).

Acetylshikonin-derived intracellular ROS generation in HCT-15 and LoVo cells was quantified as mean fluorescence intensity (MFI) by FACS using DCF-DA, permeable fluorescent, and chemiluminescent probes. Treatment with acetylshikonin induced a significant (*p* < 0.05) increase of intracellular ROS generation in HCT-15 and LoVo cells. ROS level was increased by 24.1%, 55.7%, and 72.6% (*p* = 0.0002) in HCT-15 cells and 105.1%, 188.8%, and 197.7% (*p* = 0.011) in LoVo cells, after 6 h of treatment with 1.25, 2.5, and 5 *μ*M acetylshikonin, respectively (Figures 5(a) and 5(b)). The ROS generation was reduced by 34.4% (*p* = 0.0127) in HCT-15 cells and 123.3% (*p* = 0.0094) in LoVo cells compared to cells only treated with acetylshikonin, when cotreated with NAC for 6 h (Figures 5(c) and 5(d)). The ROS generation after 6 h was immoderate after 6 h of treatment and had no discrimination; thus, we evaluated that 6 h was the most effective point to analyze ROS level in acetylshikonin-treated colorectal cancer cells. In NAC-treated cells, acetylshikonin-induced ROS generation in both cells was significantly (*p* < 0.05) abolished.

### 3.4. NAC Inhibited ROS-Mediated Apoptosis by Acetylshikonin in HCT-15 and LoVo Cells

**Figure 6:**
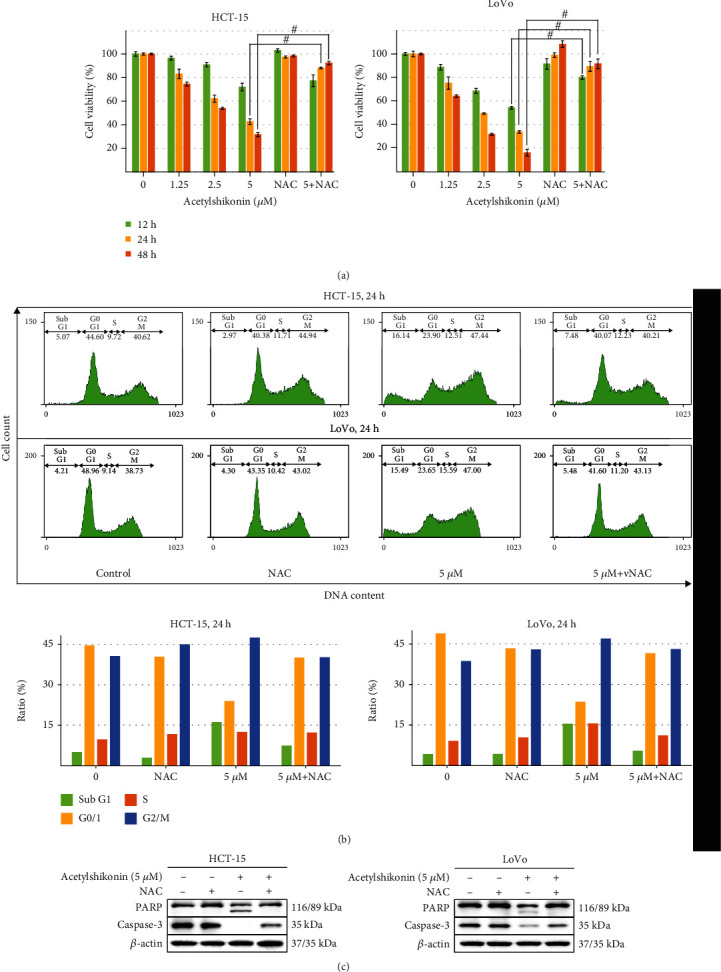
Cell viability and antiproliferative effect of acetylshikonin-treated HCT-15 and LoVo cells were assessed when cells were cotreated with NAC in control and acetylshikonin (5 *μ*M). (a) Cell viability was assessed with MTT assay. The data represent the mean ± SD of three independent experiments. Number sign indicates a significant difference of NAC-treated cells from acetylshikonin-treated cells (^#^*p* < 0.05). (b) Control cells and acetylshikonin-treated cells were incubated for 24 h with or without NAC and stained with propidium iodide (PI) for a flow cytometric analysis of DNA content. Data are represented in bar graphs. (c) Apoptosis-related protein expressions were analyzed by western blot analysis. *β*-Actin was used as a gel-loading control.

The cell viability was increased when acetylshikonin-treated cells were cotreated with NAC. The viability was increased by 5.31% (*p* = 0.4232), 45.47% (*p* = 0.0019), and 60.74% (*p* = 0.0014) in HCT-15 cells and 25.71% (*p* = 0.002), 55.65% (*p* = 0.0074), and 75.65% (*p* = 0.005) in LoVo cells at 12 h, 24 h, and 48 h, respectively (Figure 6(a)). In the cell cycle arrest assay, the result showed that portions of subG1 phase were decreased in cells cotreated with NAC. Portions of subG1 phase were reduced by 8.66% in HCT-15 and 10.01% in LoVo compared to those cells treated only with acetylshikonin. Slight increases in G2/M phases occur, but no significant distinction was observed between cells treated only with NAC or DMSO (control) (Figure 6(b)). In the western blotting result, it evidently showed a decrease of caspase-3 and PARP in acetylshikonin-treated cells and recovery in cotreated cells (Figure 6(c)). According to these results, ROS has a decisive effect in apoptosis of colorectal cancer cells, when treated with acetylshikonin.

### 3.5. Acetylshikonin Induced Apoptotic Stimulus in HCT-15 and LoVo Cells

**Figure 7:**
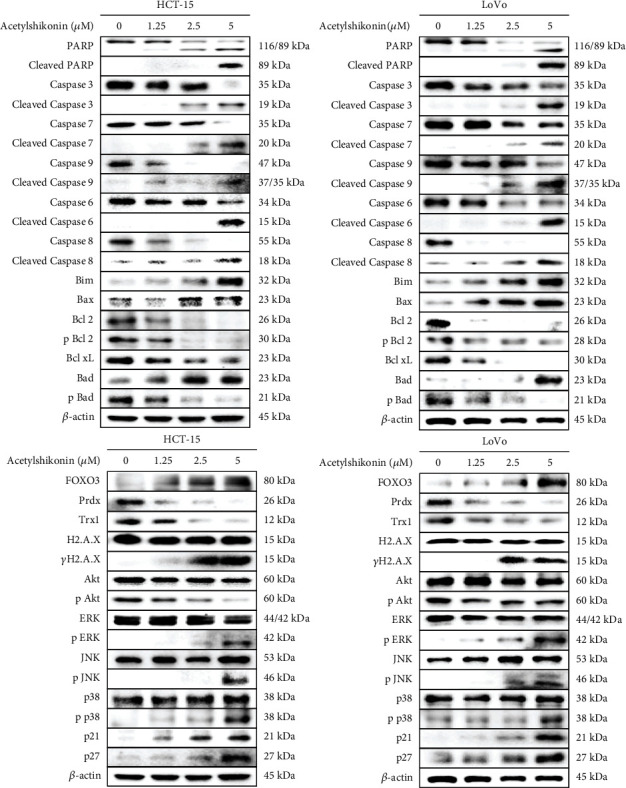
Analysis of protein expression in acetylshikonin-treated HCT-15 and LoVo cells by western blot analysis. HCT-15 and LoVo cells were treated with 0, 1.25, 2.5, and 5 *μ*M of acetylshikonin for 24 h, and apoptosis- or survival-related proteins were analyzed. *β*-Actin was used as a gel-loading control.

To investigate the apoptotic effect of acetylshikonin against colorectal cancer cells, western blotting was performed. Treatment of acetylshikonin induced cleavage of poly (adenosine diphosphate-ribose) polymerase (PARP), caspase-3, caspase-7, caspase-9, caspase-6, and caspase-8, which are important modulators of apoptosis in both HCT-15 and LoVo cells. Also, the expressions of antiapoptotic proteins peroxiredoxin (Prdx), thioredoxin 1 (Trx1), Bcl-2, p-Bcl-2, Bcl-xL, and pBad were downregulated, while expressions of proapoptotic proteins Bim, Bax, and Bad were upregulated. Moreover, the expressions of kinase proteins were altered. In acetylshikonin-treated colorectal cancer cells, the protein levels of phosphorylated mitogen-activated protein kinase (p-ERK, p-JNK, and p-p38) were increased, while protein level of pAkt was decreased. The protein levels of p21, p27, and FOXO3, which are proteins related to inhibition of cell proliferation and survival, were upregulated (Figure 7).

### 3.6. Acetylshikonin Induced Apoptotic Stimulus via MAPK Activation and Nuclear Localization of FOXO3 in HCT-15 and LoVo Cells

**Figure 8:**
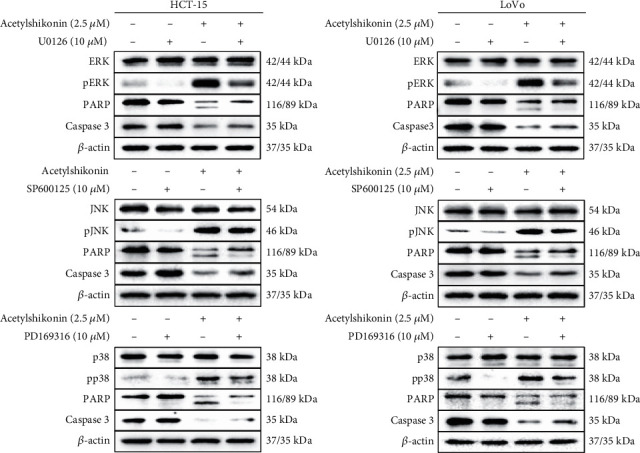
Western blot analysis of apoptotic protein expressions in HCT-15 and LoVo cells treated with MAPK inhibitors. Acetylshikonin-treated HCT-15 and LoVo cells were incubated for 24 h with or without cotreatment of MAPK inhibitors. *β*-Actin was used as a gel-loading control.

**Figure 9:**
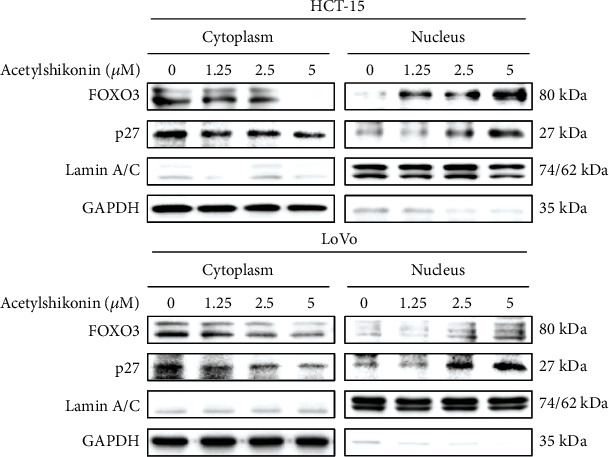
Translocation of FOXO3 and p27 proteins from the cytoplasm to the nucleus in colorectal cancer cells treated with acetylshikonin. Nuclear fractional western blotting results of HCT-15 and LoVo cells treated with acetylshikonin (0, 1.25, 2.5, and 5 *μ*M). The protein levels of FOXO3 and p27 in the cytoplasm and nucleus were analyzed by western blotting using specific antibodies. GAPDH and Lamin A/C were used as loading control proteins for the cytoplasm and nucleus, respectively.

Cotreatment of MAPK (ERK, JNK, and p38) inhibitors (10 *μ*M) in acetylshikonin-treated colorectal cancer cells had slightly inhibited cleavage of PARP and activation of caspase 3, which are key indicators of apoptosis (Figure 8). This result shows certain evidence that upregulation of MAPK in HCT-15 and LoVo is correlated to acetylshikonin-induced apoptosis. To investigate the protein location and level of FOXO3 and p27 in acetylshikonin-treated colorectal cancer cells, nuclear fractional western blotting was performed. Treatment with acetylshikonin in HCT-15 and LoVo cells for 12 h induced decrease of cytoplasmic protein levels of FOXO3 and p27 and increase of nuclear protein levels of those proteins in a dose-dependent manner (Figure 9). These results suggest that acetylshikonin has triggered the translocation of the p27 and FOXO3 proteins from the cytoplasm into the nucleus in HCT-15 and LoVo cells.

## 4. Discussion

Phytochemicals are in considerable interests as source of drug or chemotherapy development options [37, 38]. Acetylshikonin is a naturally occurring naphthoquinone, a shikonin derivative, which is noted for its inflammatory and anticancer effects [39]. The recent studies have revealed anticancer and preventive effects of acetylshikonin, such as ROS-mediated caspase activation [40], induction of cell cycle arrest via p21 and caspase-3 activation [41], and suppression of the NF-*κ*B activity [42] in various cancer cell lines. However, the anticancer effects of acetylshikonin in colorectal cancer via FOXO3 activation have not been well studied. Thus, in this study, we aimed to reveal antiproliferative effect of acetylshikonin in colorectal cancer cells and to present as an alternative candidate agent.

Cell apoptosis can be triggered by intrinsic or extrinsic pathways; intrinsic cues involve changes in mitochondria membrane permeability, and extrinsic cues involve activation of death receptors such as Fas or TNF*α*R, which both cues lead to caspase activation [43, 44]. The result from western blotting analysis shows that acetylshikonin induced activation of both caspase-8 and caspase-9 in HCT-15 and LoVo cells (Figure 7), which are the key initiators of caspase cascades in apoptotic cells [45]. Activation of caspase-8 and caspase-9 led to activation of caspase-3, caspase-6, and caspase-7 and resulted in apoptosis of colorectal cancer cells.

FOXO3 is now in a great interest in clinical research as a prognostic biomarker for cancer patients [46]. Phosphorylation of FOXO3 at Ser184 by Akt provides binding site for 14-3-3 chaperone protein, which results in delocalization from the nucleus and degradation by the ubiquitin-proteasome system [47, 48]. It was also reported that at high levels of Akt-mediated phosphorylation of FOXO3, full recovery and survival of cancer patients has a low rate [49]. In contrary, FOXO3 activity can be promoted by stress-activated MAPK (p38, JNK, and pERK) [50]. Phosphorylation of FOXO3 at Ser 574, which is a phosphorylation site of JNK, promotes localization of FOXO3 in the nucleus and transcription [51]. Also, the previous study has shown that expression of ERK and p38 proteins had induced phosphorylation of FOXO3 and increased its activity [52]. Our western blotting results (Figures 7 and 8) show that acetylshikonin treatment induced inhibition of Akt and activation of MAPK, which resulted in activation of FOXO3 protein. Also, inhibition of MAPK inhibited the apoptotic signaling in both colorectal cancer cells (Figure 8). Moreover, the nuclear fractional western blot analysis (Figure 9) has confirmed the translocation of FOXO3 to the nucleus, which leads to upregulation of series of target genes and apoptosis consequently [53]. These results show that our findings are relevant to the previous studies.

We found that nuclear translocation of p27 was also occurred by acetylshikonin treatment (Figure 9), which results in suppression of cell cycle progression and induction of apoptosis. Overexpression of cytoplasmic p27 may inhibit apoptosis in tumor cells, via mediating activation of Akt, which is a canonical suppressor protein of apoptosis, and inhibition of cytochrome c release and caspase activation [54, 55]. Despite these properties in the cytoplasm, p27 acts as an inhibitor of CDK2 when localized in the nucleus [56, 57]. According to the previous studies, nuclear localization of p27 does not just play a role in cellular apoptosis by itself but also supports nuclear translocation of FOXO3 via inhibiting activation of Akt.

FOXO3 translocation (activation) is also a critically involved in ROS accumulation and ROS-mediated apoptosis. FOXO3 regulates the expression of proapoptotic BH3-only proteins (Bim and Bad) and antiapoptotic Bcl-2 proteins (Bcl-2 and Bcl-xL), which triggers mitochondrial membrane permeabilization [58]. Our western blotting analysis results (Figure 7) show that the expressions of proapoptotic proteins were upregulated, while those of antiapoptotic proteins are downregulated in a dose-dependent manner. Moreover, the protein levels of Prdx and Trx1 were downregulated in acetylshikonin-treated cells. In Trx system, those include Prdx and Trx1 are involved in intracellular redox homeostasis regulation and signaling cascade that mediates apoptosis [59]. NAC is an antioxidant generally used in investigating roles of ROS. It inhibits activation of MAPK (JNK, p38) and several proapoptotic proteins, thus enhances cell survival [60]. Our results analyzed with NAC-treated cells show increase of cell viability in NAC-treated cells (Figure 6), which support the data that acetylshikonin-induced apoptosis is mediated by intracellular ROS accumulation. These results imply that treatment of acetylshikonin induces nuclear translocation of FOXO3 followed by ROS-mediated apoptosis signaling cascade.

**Figure 10:**
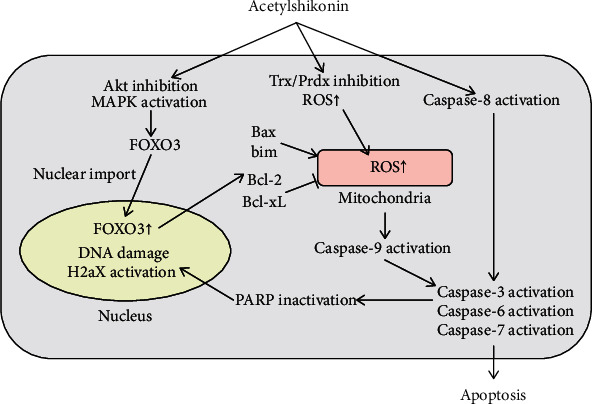
Schematic representation of the acetylshikonin-mediated apoptosis signaling pathway.

In this study, we examined the apoptotic activity of acetylshikonin against HCT-15 and LoVo cells and investigated its apoptotic mechanisms. We have revealed that the apoptotic activity of acetylshikonin in HCT-15 and LoVo cells was mediated by translocation of FOXO3 into the nucleus, which induced by Akt inhibition and activation of MAPK. This results in mitochondrial membrane permeabilization, which leads to activation of caspase cascade and apoptosis consequently (Figure 10). However, the experiments were done only in cellular level; thus, further studies must be proceeded to elucidate the anticancer effects *in vivo*. This study is believed to provide general understanding of apoptotic mechanisms of acetylshikonin in colorectal cancer and give an idea as a candidate in developing anticancer therapeutic alternatives.

## Data Availability

The authors confirm that the data supporting the findings of this study are available within the article.

## Abbreviations

TUNEL: Terminal deoxynucleotidyl transferase dUTP nick end labeling
PARP: Poly (ADP-ribose) polymerase
Akt: Protein kinase B (PKB)
FOXO3: Forkhead box O-3
Prdx: Peroxiredoxin
Trx1: Thioredoxin 1
DMSO: Dimethyl sulfoxide
FBS: Fetal bovine serum
PBS: Phosphate-buffered saline
PI: Propidium iodide
DCF-DA: 2′,7′-Dichlorofluorescin diacetate
ROS: Reactive oxygen species
NAC: N-acetyl cysteine
